# A Comparative Computer Simulation of Dendritic Morphology

**DOI:** 10.1371/journal.pcbi.1000089

**Published:** 2008-06-06

**Authors:** Duncan E. Donohue, Giorgio A. Ascoli

**Affiliations:** Neuroscience Program and Krasnow Institute for Advanced Study, George Mason University, Fairfax, Virginia, United States of America; UFR Biomédicale de l'Université René Descartes, France

## Abstract

Computational modeling of neuronal morphology is a powerful tool for understanding developmental processes and structure-function relationships. We present a multifaceted approach based on stochastic sampling of morphological measures from digital reconstructions of real cells. We examined how dendritic elongation, branching, and taper are controlled by three morphometric determinants: Branch Order, Radius, and Path Distance from the soma. Virtual dendrites were simulated starting from 3,715 neuronal trees reconstructed in 16 different laboratories, including morphological classes as diverse as spinal motoneurons and dentate granule cells. Several emergent morphometrics were used to compare real and virtual trees. Relating model parameters to Branch Order best constrained the number of terminations for most morphological classes, except pyramidal cell apical trees, which were better described by a dependence on Path Distance. In contrast, bifurcation asymmetry was best constrained by Radius for apical, but Path Distance for basal trees. All determinants showed similar performance in capturing total surface area, while surface area asymmetry was best determined by Path Distance. Grouping by other characteristics, such as size, asymmetry, arborizations, or animal species, showed smaller differences than observed between apical and basal, pointing to the biological importance of this separation. Hybrid models using combinations of the determinants confirmed these trends and allowed a detailed characterization of morphological relations. The differential findings between morphological groups suggest different underlying developmental mechanisms. By comparing the effects of several morphometric determinants on the simulation of different neuronal classes, this approach sheds light on possible growth mechanism variations responsible for the observed neuronal diversity.

## Author Summary

Neurons in the brain have a variety of complex arbor shapes that help determine both their interconnectivity and functional roles. Molecular biology is beginning to uncover important details on the development of these tree-like structures, but how and why vastly different shapes arise is still largely unknown. We developed a novel set of computer models of branching in which measurements of real nerve cell structures digitally traced from microscopic imaging are resampled to create virtual trees. The different rules that the models use to create the most similar virtual trees to the real data support specific hypotheses regarding development. Surprisingly, the arborizations that differed most in the optimal rules were found on opposite sides of the same type of neuron, namely apical and basal trees of pyramidal cells. The details of the rules suggest that pyramidal cell trees may respond in unique and complex ways to their external environment. By better understanding how these trees are formed in the brain, we can learn more about their normal function and why they are often malformed in neurological diseases.

## Introduction

Dendritic morphology underlies many aspects of nervous system structure and function. Dendrites, along with axons, define the connectivity of the brain [1],[2], and play a large role in information processing at the single cell level [3],[4]. Many studies have highlighted the importance of dendritic branching pattern in neuronal behavior. Mainen and Sejnowski [5] have shown that the full range of firing patterns for a wide variety of cortical cell types can be accounted for by branching morphology alone. Others have shown that the backpropagation of action potentials into the dendrites is strongly affected by branching pattern [6]. These results, among others, have contributed to a now widespread acceptance that dendritic morphology is an essential substrate of brain activity and function.

Despite its importance, dendritic branching remains poorly understood [7]. Dendritic branching is driven by a complex interaction of intracellular and extracellular signaling cascades which are proving difficult to completely unravel by molecular biology alone. The same chemical can have different effects in different cells [8] and even different parts of the same cells [9]. Much of the molecular work is carried out on cultured cells where separating apical and basal trees, and even dendrite from axons, is difficult (for example see [10]).

Computational modeling offers a complementary approach to traditional molecular means of uncovering fundamental properties of dendritic branching (e.g., [11],[12]). Here we focus on data driven simulations, where the parameters controlling branching behavior are measured from real cells, reduced to statistical distributions, and resampled to form virtual trees (e.g.. [13]–[16]). One advantage of this approach is the insights it gives into dendritic development. Many attempts have been made to model mechanistic aspects of dendritic development directly, such as MAP2 phosphorylation states [17], or growth cone navigation [18]–[20]. Other models, while not aiming to represent developmental processes explicitly, can yield insights into general principles or specific mechanisms at play. For example, the 3D modeling approach used by Samsonovich and Ascoli [21] demonstrates the importance of somatic repulsive forces for the shaping of principal cells in the rat hippocampus.

**Figure 1:**
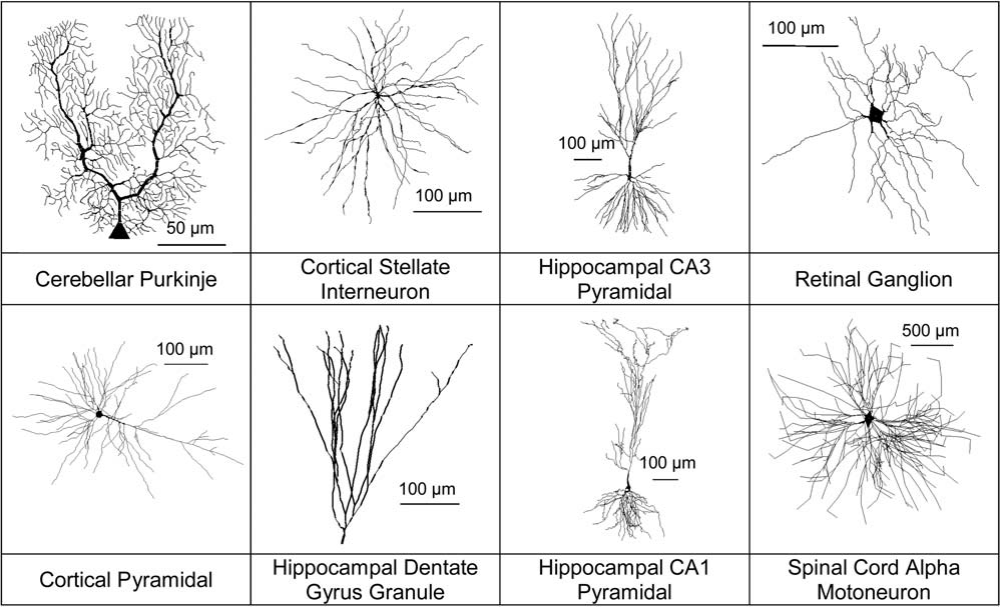
Dendritic diversity. Sample cells showing the variety of tree morphologies used as data for this study.

While data driven simulations have increased our understanding of dendritic development, they are difficult to compare directly. Different studies often focus on separate structural levels or details, and are rarely based on the same cell classes. Here we expand on previous approaches by testing a suite of three closely related models, both individually and in hybrid combinations. Also, because data driven modeling generally requires quality neuronal reconstructions, they tend to be limited to one or two dendritic tree types. From these studies, it is often difficult to determine how general the results are, and to discern biological insights from data or model peculiarities. With a large digital database of neuromorphological reconstructions now, online (NeuroMorpho.Org), we were able to apply our models to a wide variety of dendritic trees (Figure 1) from 16 different labs. This allows the separation of general trends from more specific model-morphology interactions.

**Figure 2:**
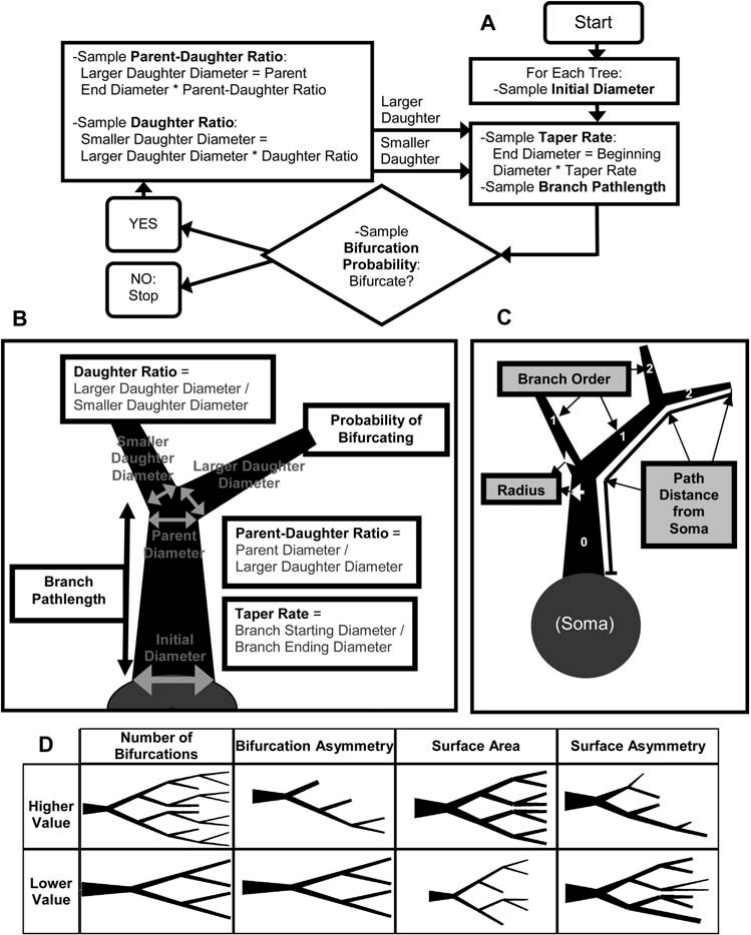
Model parameters and flow. (A) Flowchart showing how virtual trees are created from sampled basic parameters. (B) Depiction of the basic parameters. (C) Depiction of the three fundamental determinants which constrain the sampling of the basic parameters. (D) Morphometrics which are neither fundamental nor basic are emergent to the model and can be employed to compare the real and virtual trees.

The core of our modeling approach is a recursive branching process as described in Figure 2A (detailed in Materials and Methods and [22]). All of the *basic parameters* of the model (defined in the five text boxes in Figure 2B) are measured from each real cell and resampled to create virtual trees. Every branch in the real trees has an associated taper rate and pathlength, every bifurcation has a daughter diameter ratio, etc. With every basic parameter extracted from real cells, the accompanying *fundamental determinant* (Figure 2C) is also measured. For example, when measuring the taper rate of a real branch, the thickness (radius), the number of bifurcations from the soma (branch order), and the somatic path distance of that same branch are also recorded. Within each tree group (e.g., Martone's Purkinje), and for each of the three fundamental determinants, series of distributions are then generated which best describe each basic parameter for different bins of the fundamental determinant. For example, one distribution will describe all of the taper rate values which occur at Branch Order four. It is this distribution that will be sampled to select the taper rate every time a branch of order four is added to a virtual tree of this group based on this fundamental determinant (as described in Figure 2A). This process is repeated for each of the five basic parameters, 68 groups of real cells, and three fundamental determinants.

The term “fundamental determinants” is meant to describe the parameters which are primary in the model and drive the selection of other values, but should not be taken to imply that they are the only or most crucial developmental factors underlying branching behavior. The comparative approach constrains the choice of fundamental determinants to those compatible with the common mechanics of the model. Nevertheless, the chosen determinants are biologically important and have all been implicated by earlier studies (reviewed in [7]) in the control of bifurcation probability (one of our basic parameters). Radius correlates with microtubule density [23] and has previously been shown to capture some, but not all, aspects of dendritic branching in several neuronal classes [13],[22]. Branch order takes into account the division of resources from the soma and has been used to control the distribution of bifurcations in several computational models [11],[14]. Path distance affects the time of subcellular transport and signaling to and from the soma, and has been useful in constraining motoneuron and pyramidal cell virtual growth [13],[24].

**Figure 3:**
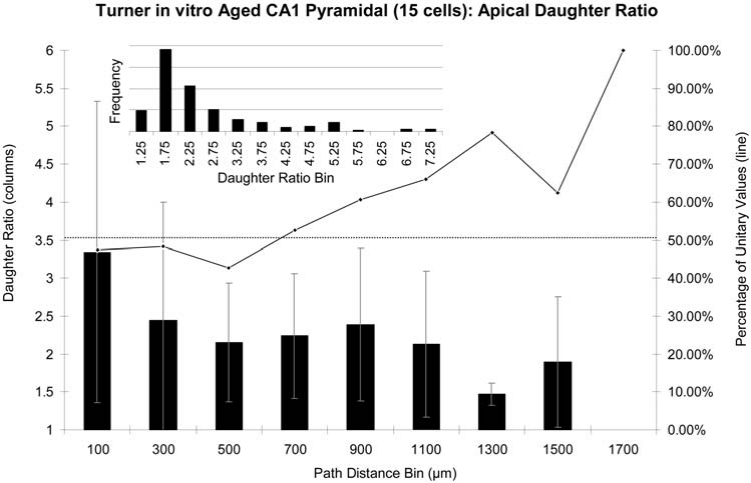
Basic parameter distributions. Example basic parameter (daughter diameter ratio) distributed irrespective of fundamental parameters (inset), as used in previous studies, and the same parameter binned by Path Distance (main plot: columns and error bars are means and standard deviations, respectively). Both the main graphs and the inset only include daughter ratio values greater than one. The solid line (secondary axis in the main plot) shows the percentage of unitary values in each bin. The dotted line represents the overall percentage of unitary daughter ratios.

In earlier efforts (e.g. [16],[25]), basic parameters were assumed to be uniformly distributed throughout the dendritic tree (Figure 3 inset). While some cell types were well captured in this way, others resulted in virtual trees which continued to bifurcate indefinitely. Later studies [22] determined that this was due to basic parameter values being applied in the virtual trees where they did not occur in the real trees. For example, in the apical trees of one group of pyramidal cells (Figure 3), the daughter diameter ratio tends to be larger near the soma than farther distally. Most importantly, the proportion of bifurcations with two equally sized daughters (unitary values of the diameter ratio) is smaller close to the soma, where most of the bifurcations occurred in this case. Without grouping by fundamental determinant, these dependencies are not captured in the virtual trees. Using radius as a fundamental determinant for all basic parameters in CA1 pyramidal cells prevented the explosive virtual growth, but the resulting trees were still excessively varied in size [22]. The model also proved to be very sensitive to radius, a notoriously noise prone measurement in neuronal reconstructions. Here we expand on this work by applying three different fundamental determinants to a wider variety of tree types.

The creation of three individual models with the same underlying mechanics also allows the implementation of hybrid variations. This step overcomes some limitations of the simpler models by introducing more freedom, but complicates biological interpretability. Most importantly, the details of how the mix models improve upon (or do not) the individual models provides information on the individual models themselves. We explored two alternatives (detailed in Materials and Methods). In the first, each basic parameter was under the control of a separate fundamental determinant (leading to 243 possible combinations). The second “Mix” strategy varied the proportional influence (in %10 steps) of each fundamental determinant in controlling all of the basic parameters.

The comparative application of different but related models to extremely diverse morphological classes enables us to look both within and across cellular/subcellular features for parameter interactions. These interactions may then point to important developmental principles. Four biologically important morphometrics which are emergent to the model are used to compare the real and virtual trees (Figure 2D, Materials and Methods). These morphometrics capture features related to both tree size and branch patterns, giving a relative measure of model behavior. A distance metric is used which takes into account both the differences between the means of the real and virtual trees, and the variability in model behavior (see Materials and Methods for details). We find that the apical and basal arborizations of pyramidal cells differ more than groups of dendrites divided by other criteria (such as tree size). We propose, based on the parameter interactions, that extracellular environment and intracellular competition for resources may be particularly important in the development of apical and basal tree types.

## Results

**Figure 4:**
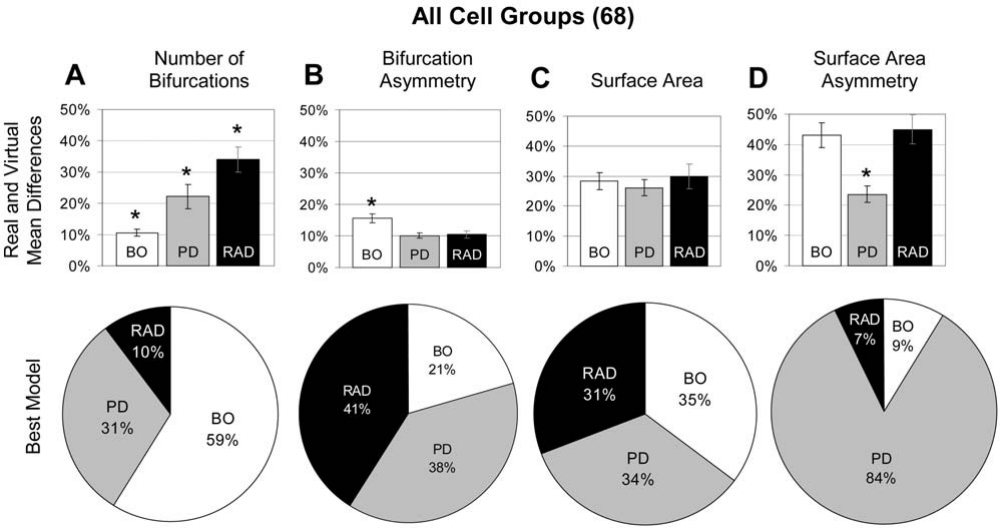
Differential ability of the three individual models to capture four emergent morphometrics. The upper portion of each panel shows the average relative difference between the means of virtual trees and those of real trees for each fundamental determinant (RAD = Radius, PD = Path Distance, BO = Branch Order). The lower portions show the proportion of times each model is the best (as measured by the distance metric) at determining the emergent morphometrics for the 68 tree groups. The fundamental determinants differ in their relative ability to capture each of the emergent morphometrics. The asterisk (*) signifies p<.05 for all figures as determined by the Mann-Whitney U non-parametric test. All error bars show standard error. (A) Number of bifurcations is best captured by Branch Order. (B) Branch Order is significantly worse than Radius or Path Distance at capturing bifurcation asymmetry. (C) Surface area is captured equally well by all three fundamental determinants. (D) Path Distance best captures surface area asymmetry.

The three individual models were evaluated in terms of their ability to produce virtual trees with values of the emergent morphometrics that best matched the corresponding real trees. Strong trends were shown when considering all of the tree classes together (Figure 4). In terms of the ability of the three fundamental determinants to reproduce the number of bifurcations across the whole set of morphologies, Branch Order was the clear winner (Figure 4A). The Branch Order model variant created trees which were significantly closer in number of bifurcations to the real trees than either Radius or Path Distance (Figure 4A upper). In particular, the mean number of bifurcations of virtual trees differed by an average of only 10% from the measured (real) value. This relative difference was over twice and nearly three times as large for the models based on Path Distance and Radius, respectively. Still looking at number of bifurcations, Branch Order was also the best model (assessed by the distance metric), for well over half of the 68 tree groups (Figure 4A lower). While a model based on branch order may be expected to best control the number of branches, apical trees of pyramidal cells offer a striking exception to this general trend, which is discussed in depth below. In this sense, the comparative approach is particularly powerful by naturally providing biologically relevant “mutual” controls among the different morphological groups and model variants.

Overall, bifurcation asymmetry was best determined by both Path Distance and Radius (Figure 4B). Both Path Distance and Radius were significantly better than Branch Order (Figure 4B upper), and were each determined to be the best for roughly twice as many tree groups as Branch Order (Figure 4B lower). No fundamental determinant was significantly better than the others at determining surface area (Figure 4C upper). Likewise, Path Distance, Radius, and Branch Order each best determined surface area for roughly one third of the tree groups (Figure 4C lower). On the other hand, surface area asymmetry was overwhelmingly best determined by Path Distance (Figure 4D). The relative difference for this emergent morphometric was on average half for the Path Distance model than for either of the other fundamental determinants. Moreover, 84% of the tree groups had their surface area asymmetry best reproduced by the Path Distance model (Figure 4D lower).

**Figure 5:**
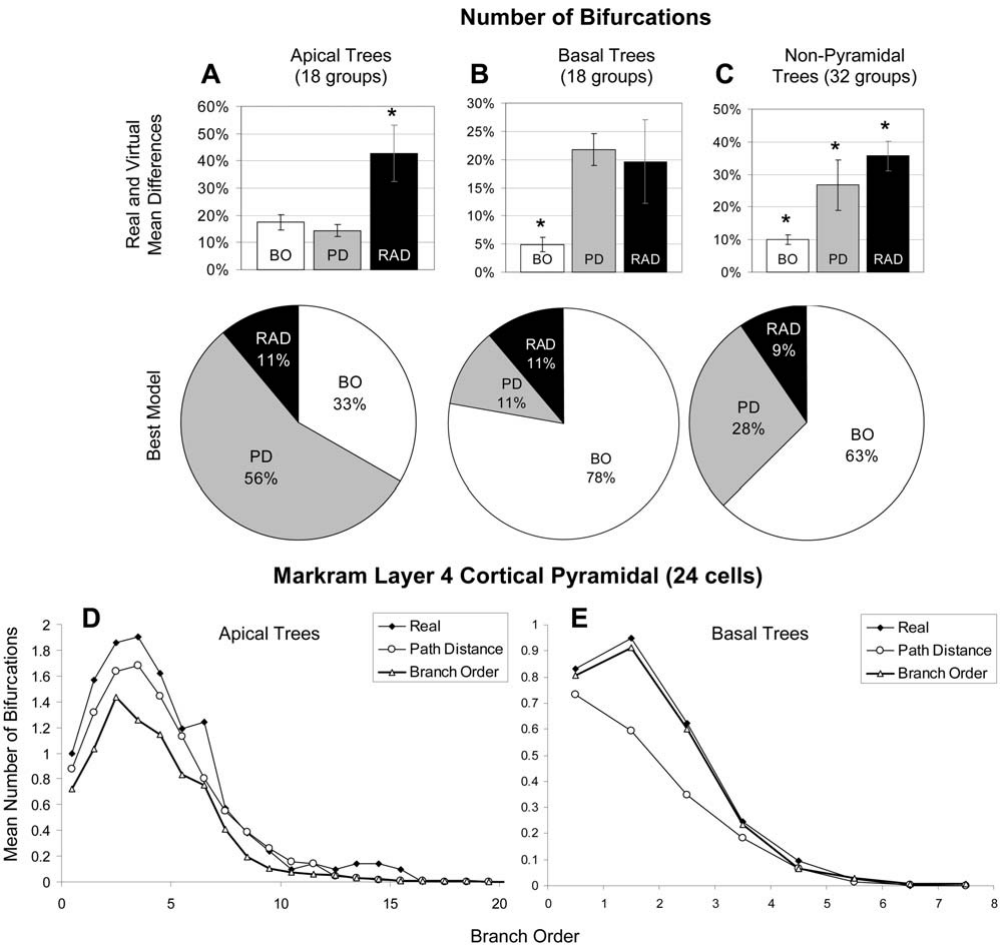
Ability of the models to capture apical and basal number of bifurcations. (A) Apical trees have their number of bifurcations best captured by Path Distance (RAD = Radius, PD = Path Distance, BO = Branch Order). (B) Basal and (C) non-pyramidal trees have their bifurcation numbers best determined by Branch Order. This may point to different underlying developmental mechanisms between apical and other tree types. (D, E) Sholl-like plots showing bifurcation number as a function of branch order for sample apical (D) and basal (E) groups of cortical pyramidal cells (Markram layer 4, N = 24). Path Distance better captures apical bifurcations while Branch Order better captures basal arbors.

These trends were generally robust throughout individual tree groups. However, a finer analysis organized by morphological classes revealed additional insights. The tree groups were first divided into apical (n = 18), basal (n = 18), and non-pyramidal (n = 32). The Branch Order model was significantly better than either Radius or Path Distance at determining the number of bifurcations in both basal and non-pyramidal tree types (Figure 5B and 5C). In particular, Branch Order “won” more than three quarters of the basal groups. This was definitely not the case for apical trees, where over half of the 18 groups had their number of bifurcations best determined by Path Distance (Figure 5A). Figure 5D shows a more detailed analysis for a representative apical tree group. In this example, Path Distance better captures not only the mean, but also the pattern of bifurcations as a function of branch order (“Sholl-like” plots). In contrast, when looking at basal trees from the same cells (Figure 5E) the Branch Order model provides a much better match to the real data.

**Figure 6:**
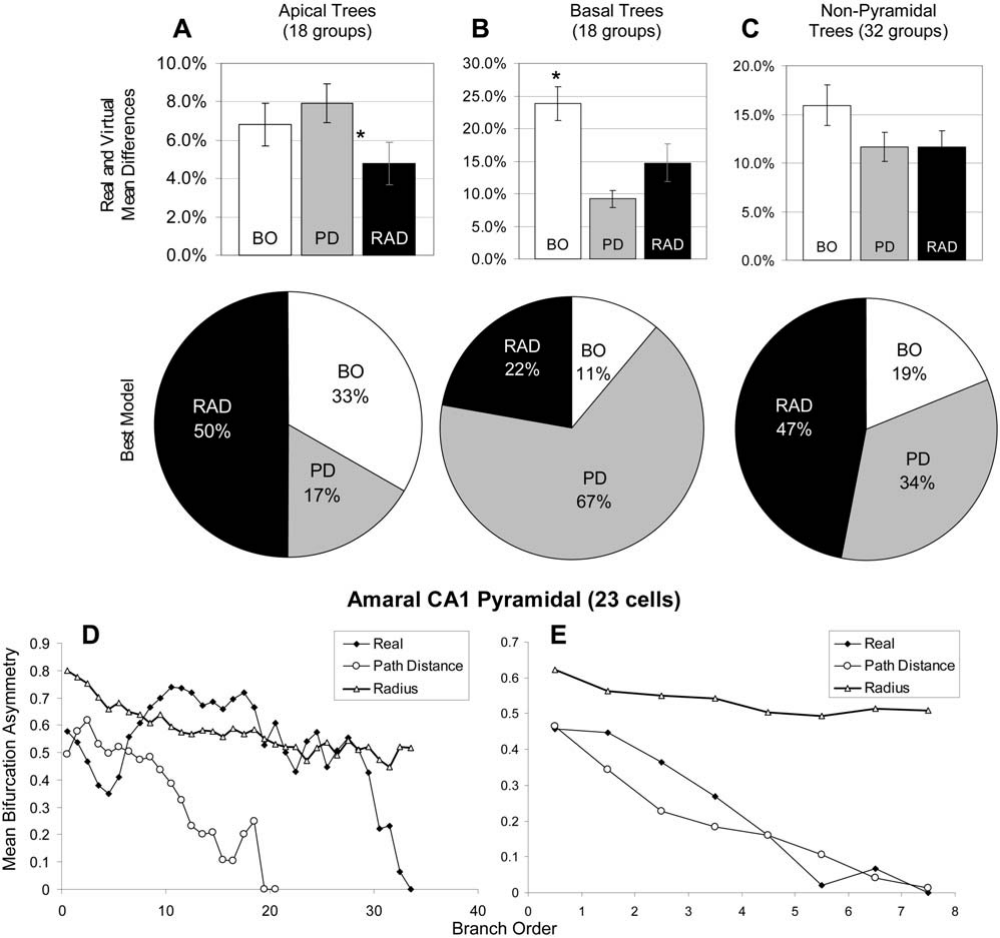
Ability of the models to capture apical and basal bifurcation asymmetry. (A) Apical trees have their bifurcation asymmetry best determined by Radius (RAD = Radius, PD = Path Distance, BO = Branch Order). (B) Basal trees have their bifurcation asymmetry best determined by Path Distance, which wins over the other two models two-thirds of the time. (C) Non-pyramidal trees lie somewhere in the middle, with neither Path Distance nor Radius giving better bifurcation asymmetry results. (D, E) The values of bifurcation asymmetry vary as a function of branch order in representative apical (D) and basal (E) groups (Amaral CA1, N = 23). Path Distance better captures the basal pattern, while the Radius model better captures apical asymmetry.

The situation is almost reversed if models are evaluated based on another emergent morphometric, namely bifurcation asymmetry instead of the number of bifurcations (Figure 6). Path Distance is the worst model at capturing apical asymmetry (Figure 6A) but the best at capturing basal asymmetry (Figure 6B), both in terms of average distance (top panels) and numbers of groups (bottom). Non-pyramidal cells fall in between apical and basal with both Radius and Path Distance producing the best results more often than Branch Order (Figure 6C). Another example Sholl-like analysis carried out on an single group of pyramidal cells is consistent with the trends observed across the corresponding sets of tree types, and opposite to the patterns observed for number of bifurcations (Figure 6D). In particular, the distribution of apical bifurcation asymmetry values as a function of branch order is better reflected by the Radius model than by the Path Distance model. Figure 6E shows that the converse is true for the basal trees from the same cells.

**Figure 7:**
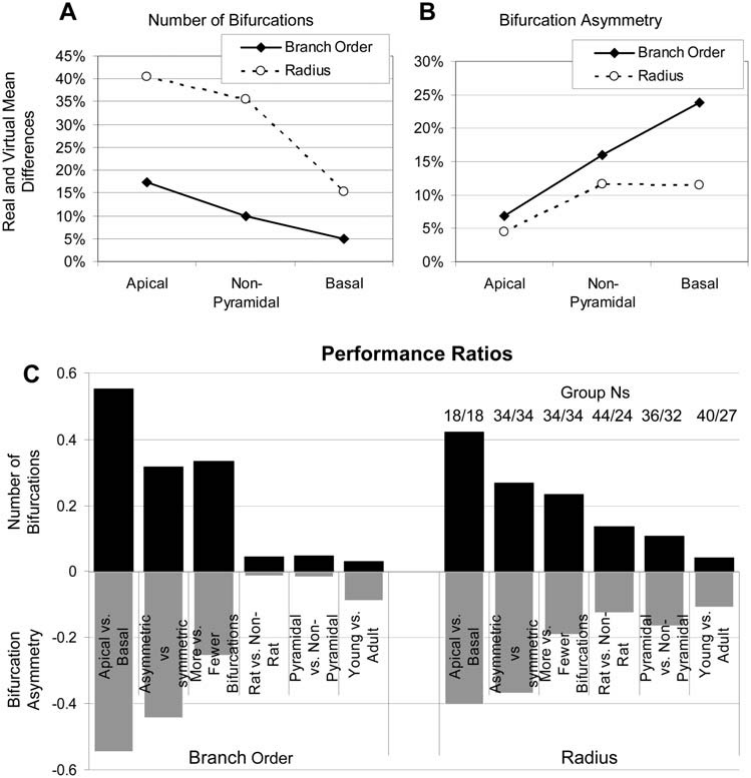
Relative magnitude of apical-basal divide. (A) Number of bifurcations is better captured by both Radius and Branch Order in basal than in apical trees. (B) Conversely, bifurcation asymmetry is better captured by both models for apical trees. In either case, non-pyramidal trees tend to lie in between apical and basal trees. (C) The relative ability of the individual models to differentiate apical from basal trees is greater than for other tree divisions. The Performance Ratio is the absolute value of the log of the ratio between the two tree types of the mean differences between real and virtual trees. Number of bifurcations is shown as positive bars (black), bifurcation asymmetry as negative bars (gray). With models based on Branch Order and Radius, the apical-basal divide shows the largest performance ratios for both bifurcation number and asymmetry. The numbers above the Radius columns represent the count of tree groups for the corresponding divisions.

While Figures 5 and 6 show that the interaction between fundamental determinants and emergent morphometrics is different for apical and basal trees, it is important to notice that the overall quality of the simulations is different as well, as becomes apparent when the units are on the same scale (Figure 7). Both Branch Order and Radius are better able to capture the number of bifurcations in basal than in apical arbors (Figure 7A), but the inverse relation holds for bifurcation asymmetry (Figure 7B). In both cases, non-pyramidal cells fall in between. This differential performance can be quantified for a given fundamental determinant and emergent morphometric as the ratio of the larger over the smaller of the mean differences between real and virtual trees for the two arbor types. In particular, we formalize the *performance ratio* as the absolute value of the logarithm of this value (this definition yields a positive value that is independent of the numerator vs. denominator). This value is larger for Branch Order and number of bifurcations and smaller for Radius and asymmetry, i.e. the contrast between apical and basal trees is greatest when testing the Branch Order model for number of bifurcations.

Such a measure also allows the comparison of different criteria to divide neuronal groups besides basal and apical, such as other cellular classifications (e.g. pyramidal and non-pyramidal), developmental stage (young and adult), animal species (rat and others), or median-based metrics (with respect to e.g. size and symmetry). The ability of the different models to differentiate between apical and basal trees is much greater than for other divisions tested (Figure 7C). In fact, at least part of the effect observed in other division may simply reflect the apical/basal divide. For example, basal trees tend to be among the smallest and most symmetric, while apical trees tend to be relatively large and asymmetric (Table 1). The contrast between the basal-apical distinction and all others is particularly prominent considering the logarithmic relation in the *performance* definition. Attempts to investigate further distinctions by cluster analysis (not shown) confirmed these observations. When clustering the 68 groups on the ability of the models to capture the emergent morphometrics, the more distant clusters break along the apical-basal-non pyramidal lines as opposed to other morphometrics (e.g. tree size, asymmetry) or metadata (e.g. animal age or strain).

**Table 1:** The 68 tree groups with the number of cells and trees in each.

Tree Type	Lab	Cells	Trees	Bif #	Bif Asym	Surf Area (μm^2^)	Surf Asym
CA1 Apical	Amaral	23	30	46.20	0.60	8053	0.53
	Claiborne	7	8	44.00	0.57	36787	0.49
	Guylas	18	18	49.61	0.61	12553	0.56
	Larkman	6	7	38.43	0.59	18074	0.52
	Turner in vivo young	24	25	46.16	0.59	18362	0.57
	Turner in vitro aged	15	18	62.50	0.57	24268	0.55
	Turner in vitro young	10	12	50.58	0.52	22053	0.54
CA1 Basal	Amaral	23	77	7.77	0.35	1340	0.27
	Claiborne	7	24	7.75	0.36	6927	0.23
	Guylas	18	62	6.85	0.35	1257	0.23
	Larkman	6	35	6.31	0.40	2297	0.40
	Turner in vivo young	24	75	10.08	0.42	3641	0.33
	Turner in vitro aged	15	48	9.52	0.41	3516	0.34
	Turner in vitro young	10	33	8.06	0.39	3269	0.35
CA3 Apical	Amaral	24	42	22.86	0.51	6504	0.46
	Barrionuevo	8	11	24.91	0.50	9188	0.48
	Jaffe	6	6	26.33	0.48	26699	0.40
	Turner	18	23	21.13	0.50	15844	0.41
CA3 Basal	Amaral	24	99	9.03	0.39	1714	0.26
	Barrionuevo	8	33	7.21	0.36	2281	0.24
	Jaffe	6	19	7.84	0.40	5966	0.25
	Turner	18	61	10.46	0.38	6771	0.34
Cortical Pyramidal Apical	Markram layer 2/3	37	43	14.35	0.50	4094	0.48
	Markram layer 4	24	21	11.43	0.50	3593	0.54
	Markram layer 5	22	23	57.43	0.60	17701	0.61
	Wearne local young	20	20	17.85	0.51	3381	0.47
	Wearne local old	17	17	18.59	0.47	5053	0.48
	Wearne long young	24	24	22.88	0.49	3282	0.47
	Wearne long old	19	19	17.74	0.48	3495	0.48
Cortical Pyramidal Basal	Markram layer 2/3	37	167	3.44	0.33	887	0.29
	Markram layer 4	24	114	2.77	0.30	854	0.47
	Markram layer 5	22	143	3.13	0.38	942	0.39
	Wearne local young	20	108	3.51	0.35	716	0.29
	Wearne local old	17	96	3.90	0.28	722	0.28
	Wearne long young	24	152	3.96	0.34	839	0.32
	Wearne long old	19	122	3.70	0.31	751	0.31
Dentate	Claiborne	43	73	8.89	0.38	11518	0.20
Gyrus	Turner in vivo	19	37	8.32	0.43	4304	0.33
Granule	Turner in vitro	19	38	6.92	0.43	4072	0.37
Cortical Interneuron	Guylas calbindin	18	69	2.78	0.30	2119	0.26
	Guylas cck	14	61	4.15	0.30	8368	0.25
	Guylas calretenin	29	83	2.64	0.26	1600	0.23
	Guylas parvalbumin	20	88	2.64	0.31	4683	0.15
	Jaffe lacunosum-mol.	13	53	3.91	0.32	3879	0.32
	Jaffe radiatum	13	50	4.40	0.43	3928	0.33
	Jaffe other	17	68	4.50	0.40	2485	0.33
	Markram	23	139	2.58	0.34	784	0.30
	Turner	13	43	4.63	0.41	2222	0.43
Purkinje	Martone	4	5	282.20	0.50	10352	0.50
	Rapp	3	3	435.33	0.50	45679	0.54
Spinal Motoneuron	Ascoli p3	9	59	11.69	0.46	4024	0.47
	Ascoli p11	8	65	9.06	0.44	1608	0.44
	Burke	6	69	13.77	0.47	54717	0.51
	Cameron 1–2 day	10	56	3.09	0.41	2471	0.32
	Cameron 5–6 day	12	83	2.08	0.31	2652	0.28
	Cameron 14–15 day	14	47	2.81	0.39	3747	0.31
	Cameron 19–25 day	8	82	2.33	0.28	1922	0.31
	Cameron phr 2 week	5	63	3.76	0.36	5791	0.32
	Cameron phr 1 month	6	66	3.36	0.33	6943	0.30
	Cameron phr 2 month	5	56	6.11	0.40	11382	0.39
	Cameron phr 1 year	6	62	6.66	0.40	27434	0.40
	Fyffe alpha	8	89	7.45	0.41	25796	0.41
	Fyffe gamma	4	29	3.48	0.37	14513	0.24
Retinal Ganglion	Miller small simple	16	60	3.07	0.34	494	0.32
	Miller small complex	5	38	7.21	0.49	2152	0.39
	Miller medium simple	15	10	14.00	0.47	704	0.48
	Miller med. complex	25	122	9.47	0.46	1291	0.39
	Miller large complex	4	14	12.64	0.44	3157	0.43

The left four columns show the mean emergent morphometric values for each group: number of bifurcations, bifurcation asymmetry, surface area, and surface asymmetry.

After comparing the ability of the “pure” fundamental determinants to control virtual growth and the emergence of various morphometrics in different cell classes, we examined the effect of mixing the influences of Branch Order, Radius, and Path Distance in the hybrid models. The “% Mix” model combines the three fundamental determinants in each of 66 fixed proportions, and samples the basic parameters according to the respective weights. In the “243 Mix” model, every basic parameter can be controlled by a different fundamental determinant. For any tree group and emergent morphometric, the best individual variants of each of these two hybrid models are singled out. Even if all variants were statistically equivalent in their ability to reproduce the morphology of real trees, better quality can be expected because of the sheer number of repetitions (and the selection of the winner). Thus, in order to compare the two hybrids and the best individual models fairly, each of the three approaches was “normalized” to the same number of 243 iterations (with varying random seeds), and the best result was chosen in each case.

**Figure 8:**
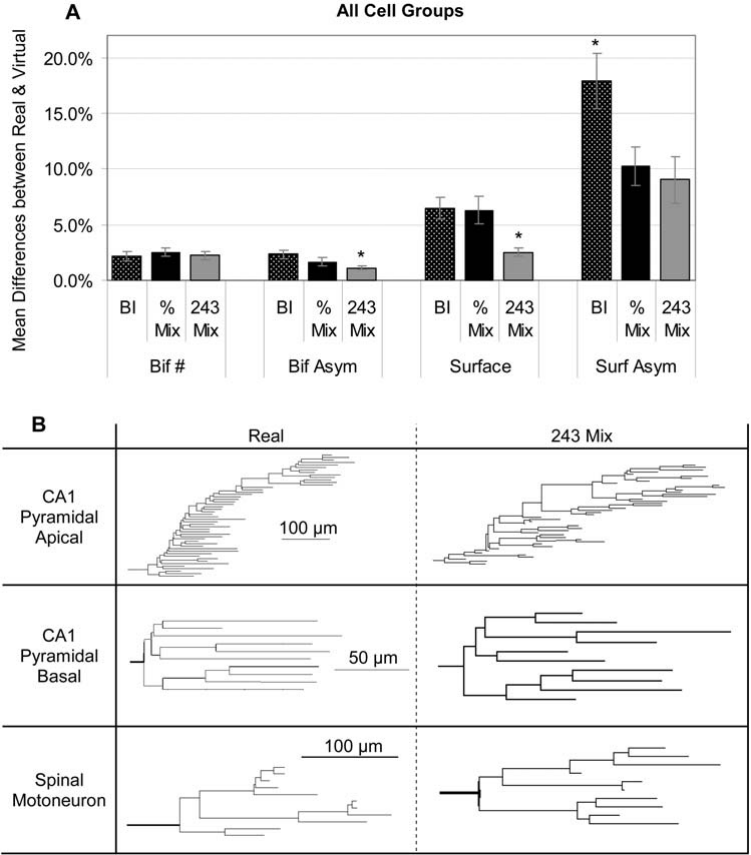
Mix model results. (A) The ability of the different model variants to capture the emergent morphometrics. The best individual (BI) and percent mixing (% Mix) were repeated with different random number seeds until they produced 243 virtual tree groups for every real one to match the number produced in the determinant mixing paradigm (243 Mix). The determinant mixing paradigm, where the sampling of each basic parameter could be controlled by a separate fundamental determinant, was significantly better at capturing bifurcation asymmetry and total surface area. Both mixing paradigms were better than the best individual models at capturing surface area asymmetry. (B) Sample real and virtual dendrograms using the determinant mixing paradigm. Scale bars are the same for each real-virtual pair.

The general trend across all 68 cell groups is that the 243 Mix clearly outperforms the best individual model, with the % Mix yielding somewhat intermediate results depending on the emergent morphometric (Figure 8). In particular, the 243 Mix is significantly better at capturing bifurcation asymmetry, surface area, and surface area asymmetry than the individual models (Figure 8A). The percent Mix paradigm constitutes an improvement relative to the best individuals with respect to bifurcation and surface asymmetry, but only for the latter significantly. In all cases, the difference between real and virtual trees was considerably larger for the surface area morphometric than for the number of bifurcations. Visual and qualitative inspection of corresponding virtual and real dendrogram confirmed these findings. In particular, the 243 Mix model demonstrated a striking ability to capture the peculiarities of dendritic branching for each of the examined tree types (Figure 8B).

**Figure 9:**
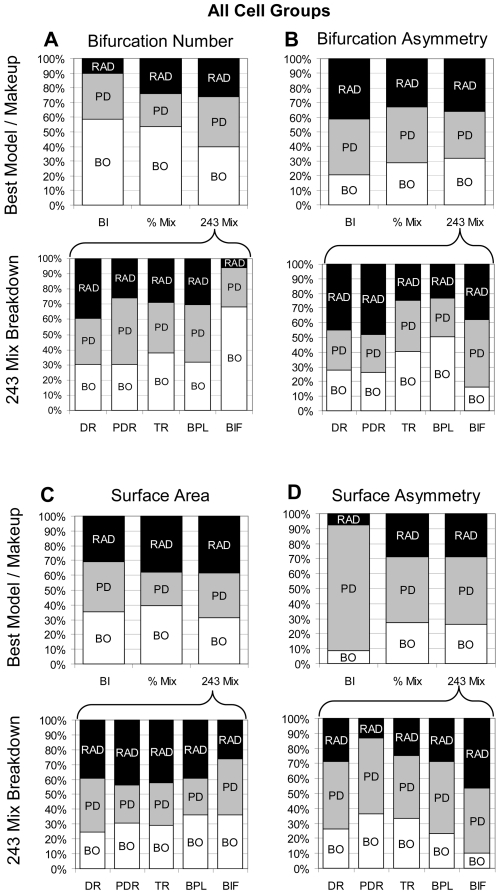
Relative contributions of the three fundamental determinants to the best models. The top row compares the percentage of winning best individual models (BI) to the relative contribution of the three fundamental determinants (RAD = Radius, PD = Path Distance, BO = Branch Order) to the winning models in the percent mixing (% Mix) and determinant mixing (243 Mix) paradigms for (A) number of bifurcations, (B) bifurcation asymmetry, (C) surface area, and (D) surface area asymmetry. The bottom row shows how the fundamental determinant contribution to the winning 243 Mix model breaks down by basic parameter (DR = daughter-ratio, PDR = parent-daughter-ratio, TR = taper rate, BPL = branch path length, BIF = bifurcation probability). The overall trend in the determinant mixing paradigm is for a more even distribution of fundamental determinant influence than seen in the best individual and percent mixing paradigms. The basic parameters with fundamental determinant weights close to those seen in the best individual model are likely the strongest drivers in the best individual model selection.

The relative weights of the fundamental determinants in the winning combination of the two hybrid models for each emergent morphometric reflects the trends observed when examining the performance of the pure models. Specifically, we compared the fraction of tree groups “won” by each individual determinant with the proportions of the winning % Mix model and the composition of the 243 Mix. Averaging the results over all tree types reveals similar values of the three determinants from the three protocols within any one morphometric property (Figure 9). Similarly, the separate examination of basal and apical arbors consistently reproduces the findings of Figures 4 and 5 (not shown).

Sampling each basic parameter using a separate fundamental determinant, the 243 Mix model provides an opportunity to gain additional insights into how specific aspects of dendritic structure and development can interact to produce mature morphologies. In particular, it is instructive to analyze how the makeup of the 243 hybrid breaks down for the five basic parameters across the emergent morphometrics throughout all cell types (Figure 9, bottom panels). For example, Branch Order controls over two thirds of the bifurcation probability in the winning variant selected by the number of bifurcations, but less than one sixth in the model that wins according to bifurcation asymmetry (Figure 9A and 9B).

When capturing bifurcation asymmetry, Branch Order contributes above average to taper rate and branch path length, Radius to daughter ratio and parent-daughter ratio, and Path Distance to bifurcation probability (Figure 9B bottom). Interestingly, Surface Area requires a finely balanced contribution of the three determinants in all five basic parameters (Figure 9C), and this emergent morphometric is particularly challenging for the other models (Figure 8A). Even though the Radius model is very rarely the best at capturing surface asymmetry, Radius is the best driver of bifurcation probability in the 243 Mix nearly half of the time (Figure 9D). These findings help to explain the success of the 243 Mix model while giving insights into which fundamental parameter/basic parameter interactions are driving the best individual model choices. For example, the best individual model with regards to the number of bifurcations seems to be highly influenced by bifurcation probability (Figure 9A). In contrast, the large percentage of tree groups which have their surface area asymmetry best captured by Path Distance may be due to the inability of Radius to determine parent-daughter ratio and of Branch Order to determine bifurcation probability with regards to this emergent parameter (Figure 9D).

## Discussion

Dendritic development is a complicated process (reviewed in [7]). Intracellular transport [26],[27], extracellularly initiated signaling cascades (e.g. [10],[28]), synaptic activity [29], membrane tension [30], and electrical activity [31] all interact to influence dendritic branching. Morphological modeling constitutes a powerful tool to try and tease out the relative influence of different mechanisms in determining the shapes of different types of dendritic trees. Theories and hypotheses about developmental principles, such as directly relating branch behavior to microtubule density [23], can be tested quantitatively and rigorously with data driven models (e.g., [22]). This is an iterative process whereby model failures can point to specific gaps in our understanding, driving new theories, experiments, hypotheses, and computational simulations.

Most previous modeling attempts varied widely in both their core methodology (i.e. the specifics of the algorithm and the choice of variables) and in the cell classes they attempted to recreate (see [7] for review). This has made direct comparison of results, and the definition of universal modeling “rules,” particularly difficult. Additionally, when only one model and a single dataset are used, it is impossible to differentiate which results are a function of biology and which are a function of the model details. We have addressed these challenges by applying several closely related models to a large database of different cell classes. Such an approach enabled the abstraction of broad tendencies as to which fundamental determinants best capture different aspects of morphology. In turn, examining the deviations from these general findings in specific cases may point to important developmental differences between tree types. This investigation led to the discovery of striking differences between apical and basal arbors of pyramidal cells.

The general results link individual fundamental determinants to the specific emergent morphometric they each best capture, and provide a baseline for comparing particular tree types. The number of bifurcations is best described by Branch Order and worse by Radius. Biologically, the cell may have the ability to “count” branch order locally when determining whether to bifurcate again, possibly detecting the partition of available downstream resources at each bifurcation. The poor performance of Radius suggests that a constant taper rate relating to steady microtubule loss is not a primary mechanism to limit or arrest branching. However, Radius is a better performer than Branch Order with regards to bifurcation asymmetry. Radius may modulate asymmetry by allowing larger branches to bifurcate while their smaller sisters terminate. Interstitial branching, the formation of side branches off of existing branches, constitutes a potential biological underpinning, as it typically produces a larger diameter disparity than terminal branching (the splitting of an extending growth cone). Path Distance can also regulate asymmetry if all branches terminate equidistant from the soma (symmetric trees), or form a distal tuft of bifurcation (asymmetric trees). This may relate to the transport of intracellular messengers or reaction to localized extracellular signals. Since only Path Distance fully succeeds in capturing surface area asymmetry, Radius may be missing vital length or position dependence. Finally, the equal contribution of all fundamental determinants to surface area suggests that this emergent morphometric is not specifically constrained by any individual corresponding biological correlate.

A limitation in regards to the interpretation of results is inherent in the restricted amount of data available in each individual group of cells. This scarcity prevents the practical or statistically meaningful investigation of the branching behavior of all neuronal classes separately. Therefore our analysis concentrated on sub-groupings of the 68 unique datasets. The groups were divided based on a wide variety of criteria, including emergent parameter values, laboratory of origin, animal species and age, brain region, and arbor type (apical, basal, or non-pyramidal). In addition to investigating the relative model performance of many of these divisions by hand, the ability of all of the model variants to capture emergent morphometrics was subjected to cluster analysis. The resulting groups were systematically compared to the above divisions as well as visually inspected for other meaningful classification criteria. Of all the various tree groupings consistent with the available collection of real morphologies, the model performance was only statistically differentiated between apical and basal dendrites (Figure 7C). Apical and basal arborizations differed in the pattern (Figures 4 and 5), and the direction of their responses (Figure 7A and 7B). Several potential biological explanations merit further investigation.

One important aspect to note is that pyramidal cells, as opposed to many of the other modeled tree types, grow in a very layer specific manner (as seen graphically in Figure 8B). Both the real and virtual CA1 apical trees show a distal increase in bifurcations, corresponding to the tuft in stratum lacunosum-moleculare. In contrast, basal trees have the majority of their terminations in a relatively small window relative to the soma (see also Figure 5D and 5E). The fact that these trees are exposed to different inputs and extracellular chemicals gradients as they cross (or do not, in the case of basal dendrites) histological layers could largely explain their contrasting branching behavior. There is some indirect experimental evidence which supports this hypothesis. Baker et al. [32] have shown differential responses of pyramidal and non-pyramidal cortical cells to neurotrophin-3. Other studies have shown that basal and apical dendrites respond differently to neurotrophins (NTs), with basal response being layer specific, while apical responses are more general, perhaps due to their crossing several cellular layers [33],[34]. These previous studies, however, have applied NTs in a bath fashion and have not looked directly at the morphology of apical trees in different layers. In order to test apical layer specific responses directly, it would be necessary to vary the NTs in a layer specific manner, perhaps through genetic manipulation of different incoming pathways, and perform layer specific analysis of apical tree morphology.

The morphological response of dendrites to NTs and other chemicals is very complex (reviewed in [7]), making the generation of specific hypotheses difficult. NTs and their receptor patterns can vary with developmental time [35],[36] and activity [37]. Other studies [9] have shown uniform sub-cellular distributions for some receptors, but rapid mobility of these receptors [38]. This with problems maintaining morphological details in certain culture preparations [39] leaves open the possibility of layer specificity, at least for some cell types or developmental periods. It is also possible that while NTs are obviously important to neuronal morphology, layer specific responses to them may be mediated through other pathways. However, some intriguing results from bath application of NTs provide possible testable hypotheses. For example, supposes it is the layer specific responses to NTs that is limiting basal dendrites to particular cortical layers. Then our results would suggest that by increasing expression of BDNF in layer 5, basal dendrites from cells in layers 4, which respond very strongly to BDNF [33],[34], may grow into that deeper layer. Also, layer 6 basal dendrites are inhibited by NGF and BDNF while layer 4 and 5 dendrites have the opposite response. Likewise apical trees in layer 6 have the weakest response to these two NTs. If pyramidal dendritic NT response is layer as well as cell type-specific, as our data suggests, the expression pattern of these NTs may be similar, and different in layer 6 than in 4 and 5. The strongest responses to NT-4 are seen in basal dendrites from neurons in layers 5 and 6, and apical dendrites from layer 4 [34]. As these three structures have no overlap in the layers they innervate, it is possible that NT-4 may provide a general growth control in these structures without disrupting layer specific responses.

An alternative or additional mechanism that could underlie the differential performance of various models in the simulation of apical and basal trees involves shifting competition for an intracellular signal or cytoskeletal metabolite. Previous statistical analyses have provided convincing indication that dendritic branching may be homeostatically regulated by global and local competition for limited intracellular resources [40]. Such an explanation could account for the sudden termination often observed in basal arbors, and the burst of bifurcations in apical tufts. More time-lapse studies of growing pyramidal cells could help clarify this possibility.

As flexibility is added to the models by allowing the different fundamental parameters to contribute to a single virtual tree through model mixing one would expect an improvement in the virtual emergent morphometrics. Both bifurcation asymmetry and surface area were significantly better reproduced by the 243 Mix paradigm than by either the % Mix or individual models (Figure 8A). However, neither mix paradigm was better than the best individual model in capturing number of bifurcations (Figure 8A), suggesting that the total branch count may be under relatively simple biological control relative to the other emergent morphometrics.

There are several dimensions in which this work could be expanded. While we are trying to gain developmental insights, digital reconstructions of real cells in publicly available databases are currently limited to adult (or at least relatively mature) neurons [41]. Based on early proposals based on electron microscopy [23], several studies, including the present one, have attempted to correlate branching behavior with local diameter (e.g. [13],[22]). However, the thickness of dendrites changes during development, and the “final” diameter measures (as reported in the digital reconstructions of real neurons) only indirectly reflect the values at the actual time of growth. With developmental time series of reconstruction data, we could model the development of dendrites more directly.

This study raised the possibility that apical and basal dendrites differ from each other due to the histological environment through which they extend, while the morphologies of non-pyramidal cells might be more intrinsically driven. By expanding the suite of fundamental determinants to include planar and radial distance from the soma, this hypothesis could be tested more directly. Such an extension would require 3D embedding of the virtual cells (see e.g., [21]). Additionally, while we have concentrated here on “normal” cells, this comparative method could also be used to detect differences between experimental preparations or disease states, possibly hinting at the underlying developmental processes.

As they occur in different parts of the same cells, the striking contrast between apical and basal trees may be costly to control and achieve, and is likely to be relevant from the information processing standpoint. This puts renewed emphasis on the question of what this divide could facilitate in the brain. Due largely to methodological considerations, the relatively thin basal branches are seldom investigated in electrophysiological experiments. Even modeling studies tend to concentrate on different divisions of the apical tree (e.g. [6],[12]). This study emphasizes the unique aspects of pyramidal cell morphology and provides motivation for a closer look at the functional consequences of its distinct arborizations.

## Materials and Methods

In this study, morphometric parameters that control dendritic branching are measured from groups of real cells and resampled stochastically to create virtual trees of the corresponding class. The real neurons consist of 736 digital reconstructions from 16 different labs. The apical and basal trees of pyramidal cells are treated separately, summing up to a total of 68 individual groups (Table 1). These 3D reconstructions were downloaded from the NeuroMorpho.Org inventory [42] in their “standardized form.” In particular, all cells are checked for format uniformity and data integrity through a combination of automated, semi-automated, and manual methods, addressing common reconstruction issues. Every morphological file (in “SWC” format) contains one numbered line for each tracing point in the neuronal structure, described by the three coordinates of its spatial position, local dendritic radius, and the number of the line representing the parent point towards the soma [43].

Virtual trees in the form of dendrograms are generated with a simple recursive algorithm (Figure 2). Starting from an initial diameter, a branch grows for a certain path length and tapers its thickness. Then it either stops or bifurcates into two daughters whose initial diameters are determined based on the parent's. Each daughter iterates independently through the same process, until all branches are terminated (Figure 2A). Thus there are five “basic” parameters controlling growth in addition to the initial diameter: branch path length, taper rate, bifurcation probability, parent-daughter ratio (between the parent diameter and the larger daughter diameter), and daughter ratio (between the larger and smaller daughter diameters). Each of these basic parameters is sampled stochastically from statistical distributions derived from the values measured in the real trees (Figure 2B).

Except for the “unique” case of the initial diameter, the basic parameters extracted from different portions of real trees vary considerably [22]. To obtain distributions faithful to the observed data, basic parameters are thus sampled according to the local value of a “fundamental” determinant. Three variants of this model are based on distinct fundamental determinants (Figure 2A), namely branch radius, path distance from the soma, and branch order (i.e., the number of bifurcations towards the soma). Thus, basic parameters measured from a homogeneous group of real dendritic trees are binned by the corresponding local value of the fundamental determinant. Branch pathlength, daughter ratio, and taper rate are based on the fundamental determinants value at the beginning of a branch, while bifurcation probability and parent-daughter ratio are based on the values at the end of branches.

Aside from the bifurcation probability (a scalar fraction), each bin is then fitted by least square error to the best of three 2-parameter functions: gamma, Gaussian, and uniform. In a previous study [22], a variety of functional distribution and fitting methodologies were tested, including reproducing all discrete values in a large lookup table for each basic parameter. As long as the basic parameter varied with the fundamental determinant, the model proved to be very robust to binning and distribution fitting particulars. Thus, the selection of parametric functions in the present work optimally combined accuracy and simplicity. For the parameters controlling diameter change, the proportion of measures assuming a unitary value (i.e. reflecting a lack of diameter change), referred to as “Unity Fraction” in previous work [22] are sampled separately according to their occurrence in each bin.

Two types of hybrid models were also tested by “mixing” the fundamental determinants. In the “% Mix” model, each fundamental determinant contributes a percentage of influence over the sampling of the basic parameters. These percentages are varied for each fundamental determinant from 0% to 100% at 10% increments. For example, Branch Order may contribute 10%, Path Length 70%, and Radius the remaining 20%. This sums up to 66 distinct variants of the % Mix model including the “pure” (unmixed) models. For the basic parameters controlling diameter, the probability of sampling a value of one is first computed as the weighted average of the three individual probabilities. For all basic parameters not determined to be one, values are sampled from all three fundamental determinant distributions and averaged together based on their relative weights. In the second mixing method, each basic parameter depends on a different fundamental determinant. For example, taper rate could be based on Radius, parent-daughter ratio on Path Distance, and bifurcation probability, branch path length, and daughter ratio all on Branch Order. With five basic parameters and three fundamental determinants, this creates an additional 3^5^ (minus the three “pure” cases) variants of this model (hence the name “243 Mix”). When comparing the individual and % Mix results to the more numerous 243 mix results, both the individual models and the % Mix models were run a total of 243 times with different random seeds.

Any morphometrics not directly used in the algorithm are “emergent” to the model. We chose four emergent morphometrics to compare virtual and real cells, selected for their biological and electrophysiological significance (Figure 2D). The total number of bifurcations provides a measure of branching complexity. Since all considered trees are binary, this count equals the number of terminations plus one. Bifurcation asymmetry characterizes how evenly those terminations are distributed throughout the tree. It is the average over all bifurcation of (n_1_−n_2_)/(n_1_+n_2_−2), where n_1_ and n_2_ are the number of terminal tips of the larger and smaller daughter subtrees, respectively. The total surface area is a size metric, while surface area asymmetry is defined by the same expression as above, but with n_1_ and n_2_ representing the surface areas of the daughter subtrees. Mean emergent morphometric values for each group of real trees are reported in the last four columns of Table 1.

A custom java program (LNded2.0), running on a Pentium M under Windows XP, extracts the basic parameters from the real cells, fits them according to the appropriate fundamental determinants, and samples the resulting statistical distributions to create virtual dendrograms. The program then outputs the emergent morphometrics from real and virtual trees to Microsoft Excel for comparison and analysis. The code and necessary documentation for all model variants is available for public download under the ModelDB section [44],[45] of the Senselab database (http://senselab.yale.med.edu). For every model and each cell group, ten virtual trees were created for each real tree. The virtual trees were then divided into ten groups, each having the number of trees matching the real groups. The mean and standard deviation for the emergent morphometrics was computed for each group and the mean of those means and standard deviations were compared to the corresponding (single) values for the group of real cells. Both for the three individual models, and for the two mixing paradigms, a “best” model was chosen for each tree group as that with the smallest “distance” between real and virtual trees. The distance metric was defined for each emergent morphometric as to account for both the gap between the real and virtual mean measures, and the stochastic variability of the simulation repeats. In particular, this metric was computed as the absolute difference between the mean of means of the ten groups of virtual cells and the mean of the single group of real cells, or as the standard error of the mean of the ten groups of virtual cells, whichever was greater.

Error bars in all figures represent standard error unless otherwise noted. An asterisk directly above a column signifies a significant difference (P<.05) from the other two columns while an asterisk between two columns signifies a significant difference only between those two columns as determined by the Mann-Whitney U non-parametric comparison using http://udel.edu/˜ mcdonald/statkruskalwallis.xls by Dr. John H. McDonald. All statistics were computed using Microsoft Excel.
